# Validation of RNA Aptamer Probes to Image *Candida albicans* in Paraffin-Embedded Sections of Wistar Rat Tongue

**DOI:** 10.1055/s-0041-1735794

**Published:** 2021-10-25

**Authors:** Boy M. Bachtiar, Chatchawan Srisawat, Retno Pudji Rahayu, Retno D. Soedjodono, Silvia Arin Prabandari, Endang W. Bachtiar

**Affiliations:** 1 Department of Oral Biology and Oral Science Research Center, Faculty of Dentistry, Universitas Indonesia, Jakarta, Indonesia; 2 Department of Biochemistry and NANOTEC-Mahidol, University Center of Excellence in Nanotechnology for Cancer Diagnosis and Treatment, Faculty of Medicine, Siriraj Hospital, Mahidol University, Bangkok Noi, Bangkok, Thailand; 3 Department of Oral and Maxillofacial Pathology, Faculty of Dental Medicine, Universitas Airlangga, Surabaya, Indonesia; 4 Department of Infectious Diseases and Veterinary Public Health, Faculty of Veterinary Medicine, IPB University, Bogor, Indonesia; 5 Primate Research Center, IPB University, Bogor, Indonesia

**Keywords:** aptamer probe, *Candida albicans*, immunostaining, paraffin-embedded tissue staining, oral candidiasis

## Abstract

**Objective** This study aimed to validate the use of Ca-apt-1, an RNA aptamer, that we generated previously as a probe for immunostaining of *Candida albicans* in rat tongue paraffin-fixed tissue sections

**Material and Methods** The performance of Ca-apt-1 as a detector molecule was compared with that of anti- *C. albicans* polyclonal antibody (PcAb), which was used as a positive control. Immunostaining images were visualized by light microscopy and were analyzed by using ImageJ software.

**Results** Microscopic results demonstrated that Ca-apt-1 specifically recognized and immunostained *C. albicans* cells of rat tongue candidiasis, with a specificity comparable to that of PcAb. ImageJ analysis showed that the area (pixels) detected by Ca-apt-1 was wider than that detected by the antibody. This indicates that the binding affinity of Ca-apt-1 toward *C. albicans* was better than that of PcAb on paraffin-embedded tissues.

**Conclusion** This study demonstrates that Ca-apt-1 can be used as a probe for immunostaining of fixed tissue sections for oral candidiasis diagnosis.

## Introduction

*Candida albicans* has been recognized for its important role in some diseases of the mouth beside oral candidiasis. 1 2 3 Oral candidiasis is a superficial infection, but in severe immunosuppressed patients, invasive and life threatening systemic candidiasis may develop. Therefore, the detection of its presence in various oral tissue is important for diagnosis and necessary for appropriate treatment.

Over the past decades, aptamers have emerged as a new class of ligands that possess a high affinity toward specific targets similar to antibodies. When compared with antibodies, aptamers are more stable, have a higher affinity, are easier to synthesize, are easier to modify, and do not require experimental animals for synthesis. 4 5 As probes, aptamers have been used for various purposes, including south-western blot assays 6 and aptamer-linked immobilized sorbent assay. 7 Thus, aptamers may replace antibodies as detector molecules for different immune assay formats, including the detection of pathogenic bacteria. 7 8 9 10

Recently, by using systematic evolution of ligands by exponential enrichment (SELEX), we generated an aptamer (Ca-apt-1) against *C. albicans* . 7 This RNA-aptamer has shown the ability to specifically recognize *C. albicans* cells, offering the potential for diagnostic development. To explore broader applications of the RNA aptamer, we further tested whether Ca-apt-1 could be used as a probe for immunostaining. Hence, the goal of this study was to develop immunoassay principles by incorporating aptamers as detection affinity-binding reagents for the identification of *C. albicans* , which is an opportunistic oral pathogen. Therefore, we exploited aptamers as an alternative to antibodies that could specifically detect *C. albicans* in immunohistochemistry tests.

## Material and Methods

### Aptamers and Polyclonal Antibody

As previously described, an oligonucleotide RNA aptamer (Ca-apt-1) with the following sequence: 5′- GGGAGUCGACCGACCAGAACGAAAGACCAACGCAGCCAAACUGAAGCCCCAGUCGCCCCGUAUGUGCGUCUACAUCUAGACUCAU-3′ was used. 7

For immunostaining, biotinylated Ca-apt-1 and its polyclonal from round 11 were prepared as described previously. 7 11 As a positive control for the immunohistochemistry assay, a rabbit polyclonal antiserum, anti- *C. albicans* (ATCC 10231), obtained from the Faculty of Veterinary IPB-University, Bogor, Indonesia was used. 12 This polyclonal antibody was purified by using Antibody Purification Kits (New England BioLabs, Massachusetts, United States) according to the manufacturer's instructions. A secondary antibody staining kit, including horseradish peroxidase (HRP)-conjugated horse anti-rabbit IgG antibody and DAB peroxidase substrate solution for color development, was purchased from GBI Labs (Golden Bridge International).

### Immunostaining of Paraffin-Embedded Tissue Sections by RNA-Aptamer-Biotin and Rabbit Polyclonal Antibody Anti- *C. albicans*

Paraffin-embedded tissue blocks were obtained from archived files in the Department of Oral and Maxillofacial Pathology, Faculty of Dentistry Universitas Airlangga, Surabaya, Indonesia. The blocks consisted of untreated candidiasis and treated candidiasis on the tongue surface with epigallocatechin gallate. 13 A paraffin-embedded cat skin specimen infected with *Microsporum canis* —provided by the Primate Research Centre (PSSP), IPB University, Bogor, Indonesia (unpublished)—was also used.

Analysis of tissue sections by immunohistochemistry (IHC) was performed by following the routine laboratory protocol at PSSP, IPB University. Tissue sections were prepared, and hematoxylin and eosin (H&E) staining was performed for morphological confirmation. To do this, 5 µm thick tissue sections of the paraffin-embedded blocks were cut, de-paraffinized with xylene, and rehydrated with a gradient concentration of ethanol. To quench the activity of endogenous peroxidase, the sections were immersed in 3% H _2_ O _2_ for 30 minutes in the dark. After washing three times with phosphate buffer saline (PBS) for 5 minutes each, the sections were immersed in blocking solution, incubated at 37°C for 30 minutes, and washed again with PBS. Subsequently, tissue sections were dehydrated through graded alcohols and subjected to antigen retrieval using 0.2% Trypsin - CaCl _2_ at room temperature for 2 hours. Sections were washed three times with PBS for 5 minutes each before blocking with 10% horse serum for 30 minutes at room temperature. Then, the sections were incubated with rabbit polyclonal antibody (1:200) or biotinylated aptamer (1:200) diluted with TBS overnight at 4°C. After washing three times with 0.01 M PBS for 5 minutes each, the sections were incubated with streptavidin-HRP for biotinylated aptamers and HRP-conjugated goat anti-rabbit IgG (GBI Labs: Golden Bridge International) for PcAb at 1:200 dilution in TBS at room temperature for 40 minutes, according to the manufacturer's instructions. After washing, the slides were incubated with 3,3′-diaminobenzidine tetrahydrochloride (DAB) chromogenic solution (Sigma) and immediately washed under tap water after color development. The sections were further counterstained lightly with hematoxylin. Slides were mounted with dibutyl phthalate xylene (DPX) and then observed under a light microscope (Carl Zeiss). Image brightness and contrast were optimized with Adobe Photoshop 7.0.

### Quantification of Images with ImageJ

Images of the immunostained tissue sections were captured by using a light microscope (Carl Zeiss). Identical light intensity and exposure settings were applied to all the images taken for each tested slide. Images were captured at ×200 magnification with a 3.3-megapixel resolution, and the images were saved as TIFF files.

Further, the images were analyzed by using the NIH ImageJ software. The area of each visible cell on the slide was measured in pixels squared by outlining the cell using the region of interest (ROI) tool. The “measure” tool under the “analyse” tab opened a “results” box with the selected area of the free drawn circle. A free drawn circle was drawn around the edge of the slide by using the oval ROI tool. Subsequently, under the “Edit” tab, the area outside of the circle was removed from the analysis area using the “clear outside” option. Then, under “image” and “type,” the image was adjusted to 8-bit, with the same contrast, using the adjust >brightness >contrast function. Next, the circularity parameter was set as “0.10 to 1.00,” and an overlay was used to highlight and count the number of cells. Further, the threshold, which is in the “image” tab under “adjust,” was set to highlight the cell as black spots with a white background. After setting the threshold, the window was closed without hitting the “apply” button, and a circle was drawn again around the edge of the slide eliminating any cells on the edge that were to be excluded from the final cell count. Then, under “process” and “binary,” the option “convert to mask” was selected to convert the image to a mask. Next, the merged cells were split with a 1-pixel wide line by using the “watershed” tool. Under “analyze,” the option “analyzed particles” was chosen, and finally, “display results,” “clear results,” “summarize,” and “exclude on edges” were selected in the order mentioned. The results displayed the number of particles that fit the parameters within the image, which was used as the total cell count. The output of this analysis contains the number of pixel intensities ranging between 0 ± 255, histogram value, where 0 was very dark, indicating dark/intense staining, while 255 was very bright, indicating very little staining. The obtained data included the mean, standard deviation, and minimum and maximum values of the measurement.

### Statistical Analysis

All data are expressed as the mean ± standard deviation. The distribution of *C. albicans* immunostained by either PcAb or Ca-apt-1 was defined by analyzing the pixel intensity values (ranging between 0 ± 225) using ImageJ software. Subsequently, difference distribution of *C. albicans* between sample groups tested (treated and untreated tongue tissue candidiasis) was compared by using Student's *t* -test. Statistical significance was set at *p* <0.05. All statistical analyses were performed by using GraphPad Prism 9.0 (GraphPad Software, Inc. San Diego, CA).

## Results and Discussion

### Detection of *C. albicans* Cells from Infected, Paraffin-Embedded Rat Tongue Tissue

Previous studies have demonstrated that aptamers can be used as immunostaining probes. 14 15 The specific aim of this study was to analyze the potential of Ca-apt-1 as a probe for apta-histochemistry. Hence, this study was not intended to assess antifungal effects. 13 Instead, we observed the microscopic images of immunostained cells to assess the direct qualitative and quantitative detection of *C. albicans* in rat tongue tissue samples. Our results showed that the Ca-apt-1 aptamer probe targeting *C. albicans* -infected cells 4 could successfully detect the fungus.

**Fig. 1:**
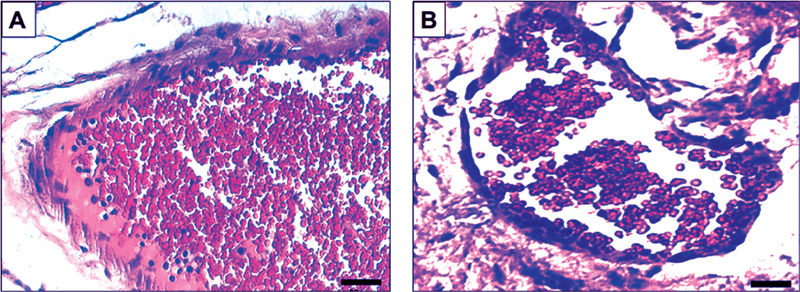
Paraffin-embedded tissue sections stained with hematoxylin and eosin for morphological confirmation. ( **A** ) Untreated rat tongue candidiasis and ( **B** ) treated tongue candidiasis in Wistar rats. Original magnification =  × 200, scale bars = 2 μM.

The Ca-apt-1 aptamer probe is useful for immunostaining of paraffin-embedded tissues, in which *C. albicans* cells are located in tissue specimens isolated from the tongue of infected rats. In this study, paraffin-embedded tissue sections of the rat tongue were prepared and probed with the biotinylated aptamer (Ca-apt-1). For comparison, the paraffin-embedded tissue sections were also probed with PcAb 12 as a standard control. The results were further verified by light microscopy, and all images were edited for brightness and contrast by using Adobe Photoshop 7.0. Thus, we first verified that *C. albicans* were present on the tongue tissue by staining the histological sections of treated (TC) and untreated (UTC) rat oral candidiasis paraffin-embedded tissues with hematoxylin and eosin (H&E). Microscopic examination of H&E-stained sections showed that in both sections, the *C. albicans* cells on the rat tongue surface had similar morphotypes, such as yeast cells and blastospores or pseudo hyphae. In general, a decreasing number of *C. albicans* cells were observed in the treated sections ( Fig. 1A and 1B ).

**Fig. 2:**
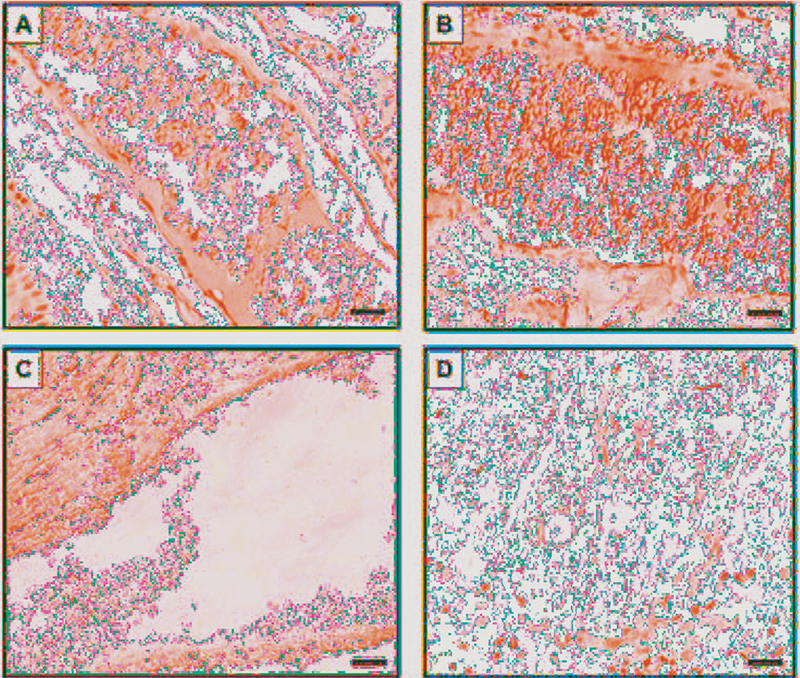
Paraffin-embedded tissue immunostaining of UTC. ( **A** ) The tissue section of UTC probed by polyclonal antibody. ( **B** ) Tissue section of UTC probed by Ca-apt-1. ( **C** ) The tissue section of UTC probed by the polyclonal aptamer. ( **D** ) Cat skin lesion sections of infected *Microsporum canis* cases probed by Ca-apt-1. All images were captured under a light microscope with ×200 magnification. Scale bars = 20 μM. UTC, untreated rat tongue candidiasis.

Next, we investigated whether the aptamer Ca-apt-1 could specifically detect *C. albicans* cells in paraffin-embedded sections. As shown in Fig. 2A and 2B , both PcAb and Ca-apt-1 immunostained the fungal cells with high specificity, with an almost similar morphotype of the *C. albicans* observed. Additionally, the pattern of the area consisting of positively- and negatively stained cells detected by Ca-apt-1 was relatively similar to that observed in cells stained with PcAb. Both probes showed a clear magenta color, therefore, indicates the presence of *C. albicans* cells. When using a polyclonal aptamer that we previously used in the SELEX selection procedure, 7 some *C. albicans* cells were detected in the paraffin-embedded tissue but were faintly stained. This result indicates that *C. albicans* cells were not specifically recognized by the polyclonal aptamer ( Fig. 2C ). Additionally, we used a sample of cat skin lesion-containing non- *C. albicans* cells, but no signal output was observed microscopically by Ca-apt-1 immunostaining ( Fig. 2D ). As expected, the aptamer probe did not cross-react with non- *Candida* cells.

**Fig. 3:**
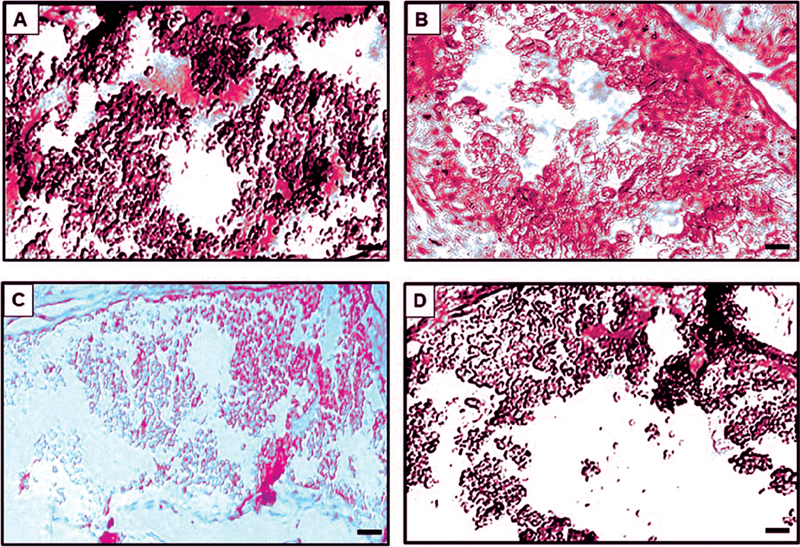
Paraffin-embedded tissue immunostaining of TC. 13 The upper panel shows the tissue section of TC probed by PcAb ( **A** ) and Ca-apt-1 ( **B** ) after a 5-day treatment. The lower panel shows the tissue section of TC probed by PcAb ( **C** ) and Ca-apt-1 ( **D** ) after a 7-day treatment; bar = 20 μM. TC, treated candidiasis. PcAb, polyclonal antibody.

**Fig. 4:**
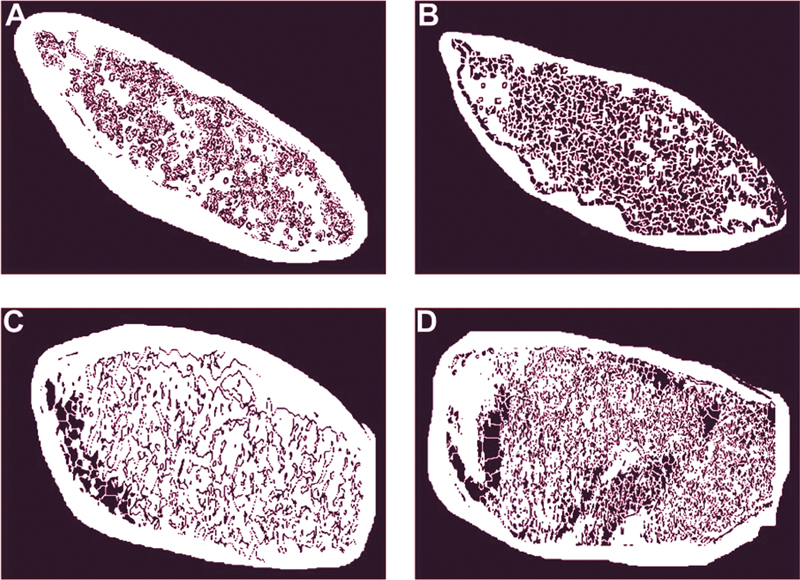
Binary image of a sample of 255 particles as black objects and white background after unnecessary parts of the images were removed. The upper and lower panels are untreated and the treated candidiasis samples, detected by polyclonal antibody ( **A, C** ) and Ca-apt-1 ( **B, D** ), respectively.

**Fig. 5:**
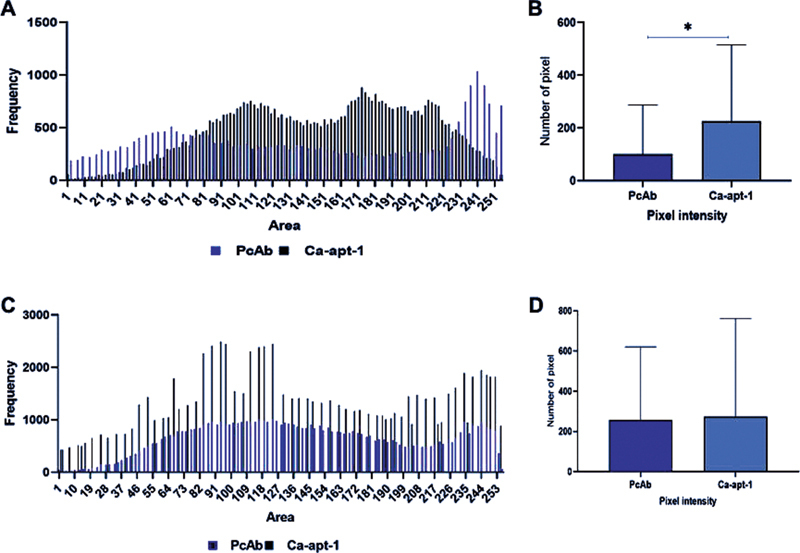
Estimated quantity of *Candida albicans* cells in the tongue of Wistar rats with oral candidiasis hybridized with Ca-apt-1 and anti- *C. albicans* polyclonal antibody. The upper panel shows the quantity of *C. albicans* cells in the tongue tissue sections of untreated oral candidiasis, and the lower panel shows the quantity of *C. albicans* cells in the tongue tissue sections of treated oral candidiasis. The left figure shows the area and frequency distribution of the probes ( **A, C** ), while the right figure shows the average intensity and number of pixels ( **B, D** ). Ca-apt-1 = RNA aptamer. The asterisk denotes a statistically significant difference. PcAb, polyclonal antibody.

Although the concept of aptamers has emerged more than a decade ago, 16 17 its clinical application in oral pathology has not yet significantly advanced. Our results demonstrated for the first time that RNA aptamer probes are useful for immunostaining *C. albicans* in paraffin-embedded tongue tissue sections. In this study, the potential of the Ca-apt-1 probe in tissue immunostaining for oral disease diagnosis was validated by testing the ability of the aptamer to discriminate the relative amount of *C. albicans* cells. First, we compared the number of *C. albicans* cells stained with Ca-apt-1 and PcAb, within the same area of tissue section of treated (for 5 and 7 days to assess the anticandidal potential of plant bioactive compounds) and untreated candidiasis reported previously. 13 The results of IHC with PcAb and Ca-apt-1 are shown in Fig. 3A–D . In all cases, strong signals were detected from both PcAb and Ca-apt-1, with a relatively similar number of *C. albicans* cells in either tissue section tested. Notably, the present study did not intend to study the histopathological features of the samples. Therefore, we further analyzed the histologic characteristics of the aptamer (Ca-apt-1) as a probe to detect *C. albicans* in the embedded tissue using ImageJ software. We analyzed the pixel score according to the histopathological features of two types of samples: Wistar rat candida infection, UTC and TC. We found that the aptamers and antibody (PcAb) exhibited a nearly identical pattern of interaction with *C. albicans* cells in embedded tissues. This result was achieved by setting the threshold tool in the imaging software according to the distribution of intensities for all cells on a given sample. Such effective interaction could be dictated by several factors, including a suitable binding affinity of either the ligand (antibody or aptamer) to its target ( *C. albicans* cells) or the expression of the immunodominant outer cell wall component of *C. albicans* 7 on the embedded Wistar rat tongue tissue. Our results showed that Ca-apt-1 bound to the targeted cells and was deposited in all regions of the embedded tongue epithelial tissue in both UTC and TC. Moreover, imaging analysis using ImageJ software revealed essentially equivalent size distribution of the observed cells. By comparing the cell distribution detected by Ca-apt-1 and PcAb, we discovered that the total sum of pixels in the UTC sample detected by Ca-apt-1 significantly exceeded those detected by the control (PcAb). As shown in Fig. 4A , there are approximately equal numbers of pixels in each of the area ranges between Ca-apt-1 and PcAb. Although Ca-apt-1 and PcAb exhibited nearly identical specificity to *C. albicans* cells, we observed that, in general, the area (pixels) detected by Ca-apt-1 was higher than that detected by PcAb ( Fig. 4B ). This may indicate that the binding affinity of Ca-apt-1 toward *C. albicans* was better than that of PcAb on paraffin-embedded tissues. 8 14 We also observed consistent qualitative ( Fig. 4A–D ) and quantitative performance of Ca-apt-1 in detecting *C. albicans* in infected tongue tissue specimens ( Fig. 5A–D ). This is in accordance with our previous study showing comparable affinity of the Ca-apt-1 probe and the monoclonal antibody in binding to *C. albicans* cells using enzyme-linked immunosorbent assay. 7 In this study, compared with anti- *C. albicans* polyclonal antibody, Ca-apt-1 showed a higher number and intensity of existing pixels. However, the cell staining pattern showed by Ca-apt-1 and PcAb was slightly different ( Fig. 5A–D ). We assumed that the aptamers and antibodies used in this study recognize different portions of *C. albicans* cells. Additionally, since aptamer provide complex tertiary, folded structures with sufficient recognition surface area, it surpasses the binding affinities of antibodies. 18

Furthermore, immunostaining of rat tongue tissue of candidiasis demonstrated that the Ca-apt-1 probe and the polyclonal antibody have very similar affinities for *C. albicans* cells, although occasional *C. albicans* cells also exhibited weak staining by the aptamer probe and the antibody, as shown in Fig. 3A–D . Since polyclonal antibodies are produced by multiple B cell clones, we assumed that the reactivity patterns of the polyclonal antibody anti- *C. albicans* is due to a combination of conserved cell surface antigens unique to *C. albicans* . 19 Therefore, the lower cell area detected by the polyclonal *C. albicans* antibody in this study is more likely due to nonspecific reactivity.

In conclusion, this study demonstrated that the RNA aptamer is useful for the immunostaining of paraffin-embedded tissues. To the best of our knowledge, this is the first report of aptamer-based detection of *C. albicans* in tongue tissues. Our work provides a clear experimental clue for the use of aptamer probes in tissue immunostaining for the diagnosis of oral candidiasis.
